# Renal cell carcinoma with an “uncoiling” tumor thrombus: intraoperative shift from level III to level IV

**DOI:** 10.1186/s12957-024-03355-z

**Published:** 2024-03-07

**Authors:** Marina Mata, Marina M. Tabbara, Angel Alvarez, Javier González, Gaetano Ciancio

**Affiliations:** 1 Department of Surgery, University of Miami Miller School of Medicine, Jackson Memorial Hospital, Miami, FL USA; 2 Miami Transplant Institute, University of Miami Miller School of Medicine, Jackson Memorial Hospital, Miami, FL USA; 3 Unidad de Cirugia Renal, Trasplante e Investigación, Hospital Ramón y Cajal, Madrid, Spain; 4 Servicio de Urología, Unidad de Trasplante Renal, Hospital General Universitario Gregorio Marañón, Madrid, Spain; 5 Department of Surgery and Urology, University of Miami Miller School of Medicine, 1801 NW 9th Ave, 7th Floor, Miami, FL 33136 USA

**Keywords:** Renal cell carcinoma, Tumor thrombus, Transesophageal echocardiography, Surgical technique

## Abstract

**Background:**

The gold standard treatment for renal cell carcinoma (RCC) with tumor thrombus (TT) is complete surgical excision. The surgery is complex and challenging to the surgeon, especially with large tumor thrombus extending into the inferior vena cava (IVC) and right atrium. Traditionally, these difficult cases required the use of cardiopulmonary bypass (CPB) with or without deep hypothermic cardiac arrest, but in recent years, different surgical techniques derived from the field of liver transplantation have been used in efforts to avoid CPB.

**Case presentation:**

We present a case of RCC with TT level IIIc (extending above major hepatic veins) that “uncoiled” intraoperatively into the right atrium after division of the IVC ligament, transforming into a level IV TT. Despite the new TT extension, the surgery was successfully completed exclusively through an abdominal approach without CPB and while using intraoperative transesophageal echocardiography (TEE) monitoring and a cardiothoracic team standby.

**Conclusions:**

This case highlights the need for a multidisciplinary approach and the utility of intraoperative continous TEE monitoring which helped to visualize the change of the TT venous extension, allowing the surgical teamto modify their surgical approach as needed avoiding a catastrophic event.

## Background

Renal cell carcinoma (RCC) is the fourth most common cancer in men and third most common in women, with an estimated incidence of approximately 81,800 new cases in the United States in 2023 [1]. RCC has a unique proclivity to extend into venous vasculature as a tumor thrombus (TT), expanding through the renal vein and inferior vena cava (IVC) in 4 to 10% of cases. The right atrium is reached by the TT in 1% of cases [2–5].

In spite of recent advances in targeted therapies and inmunotherapy, complete surgical excision remains the only curative treatment of RCC with TT, with overall survival rates up to 68% at 5 years reported in the literature [6]. Cardiopulmonary bypass (CPB) and deep hypothermic cardiac arrest have been traditionally used in the surgical management of RCC with TT venous extension into the retrohepatic inferior vena cava and right atrium (2–4, 7–8); however more recently, an exclusive abdominal approach in efforts to avoid sternotomy and CPB has been successfully utilized [5, 9–13]. However, in cases of adherent or bulky TT invading the right atrium or right ventricle, CPB is still required. The abdominal approach consist in the use of organ transplant-based techniques being the main one the Piggy-back liver mobilization to fully expose the IVC and the use of a liver self-retaining retractor to enhance exposure of the suprahepatic/infradiaphragmatic space [10–11].

We report a case of RCC with TT level IIIc (extending above major hepatic veins, but below the diaphragm; subclassification of level III tumor thrombus by Ciancio et al. [14]) that “uncoiled” during the surgery after division of the IVC ligament, transforming to level IV (TT extends into the right atrium; classification by Neves and Zincke [15]). The surgical approach for this case was exclusively abdominal using transesophageal echocardiography (TEE) monitoring; no conversion to CPB was required in spite of change in TT level.

## Case presentation

A 70-year old male with a history of hypertension, hypercholesterolemia, and hypothyroidism consulted his physician due to fatigue and dehydration after playing golf. Routine blood work showed a creatinine of 1.50 mg/dL and further workup with ultrasound revealed a right renal mass. Abdominal MRI confirmed the presence of an 8 cm right renal tumor with TT extending through the IVC above the major hepatic veins but below the diaphragm (level IIIc) [14]. The patient denied any previous episodes of fever, hematuria, dysuria, or any other lower urinary tract symptoms. CT scan of chest and abdomen showed no distant metastases; therefore, complete surgical excision of this case of RCC with level IIIc TT was the recommended treatment. This case presentation was in accordance with the University of Miami Institutional Review Board and Helsinki Declaration (as revised in 2013). The patient was informed of the risks of surgery including infection, bleeding, blood transfusions, pulmonary emboli and impossibility of complete surgical excision. Written informed consent was obtained prior to surgery.

## Procedure in detail

A modified Chevron incision was placed approximately 2 fingerbreadths below the right costal margin and extended out laterally to the mid-axillary line and medially 3-4 cm towards the left costal margin. A Thompson retractor was used in order to elevate the costal margins. The right kidney with the tumor was dissected on its lateral and posterior sides, and then mobilized medially. The renal artery was identified, ligated and divided using the posterior approach (16–17).

**Fig. 1:**
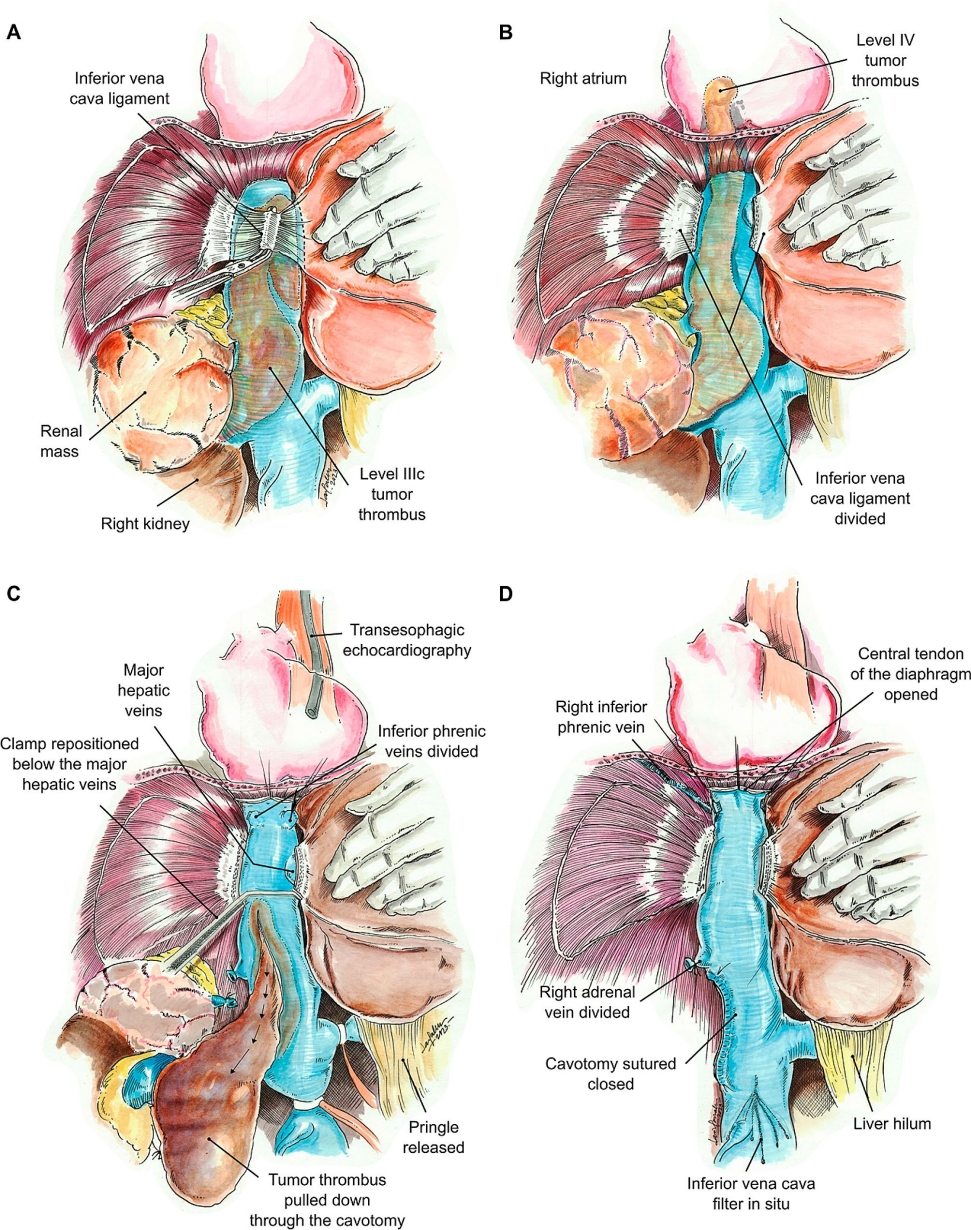
(**A**) Dissection of the IVC ligament. TT extends above major hepatic veins, but below the diaphragm (level IIIc). (**B**) After the IVC ligament is divided TT “uncoils” and now extends into the right atrium (level IV). (**C**) Under TEE guidance, the TT is pulled down to the infrahepatic IVC through the cavotomy, allowing repositioning of the vascular clamp below the major hepatic veins and releasing of the Pringle maneuver. (**D**) Cavotomy is closed and all vascular clamps are released

After renal artery ligation, the collateral circulation collapsed making the remaining dissection easier to perform. Subsequently, liver mobilization using the Piggy-back liver transplant technique [13], with ligation of the ligamentumteres, falciform ligament and left triangular ligament. The liver was mobilized off the IVC and small hepatic veins were ligated and divided in order to expose the infrahepatic, intrahepatic and suprahepatic portions of the IVC [10]. However, after the division of the IVC ligament under TEE monitoring, the TTseemed touncoil without tumor rupture and changed from level IIIc (TT extending above major hepatic veins, but below the diaphragm [14]) to level IV (TT extends into the right atrium [15]) (Fig. 1).

At this point, the surgery was stopped and the cardiothoracic team was called into the operating room to have CPB equipment and cardiothoracic surgical tray (to open the thorax) on standby. In the case that CPB was needed, the chest would have to be accessed for cannulation in the superior vena cava or right atrium and aorta.

**Fig. 2:**
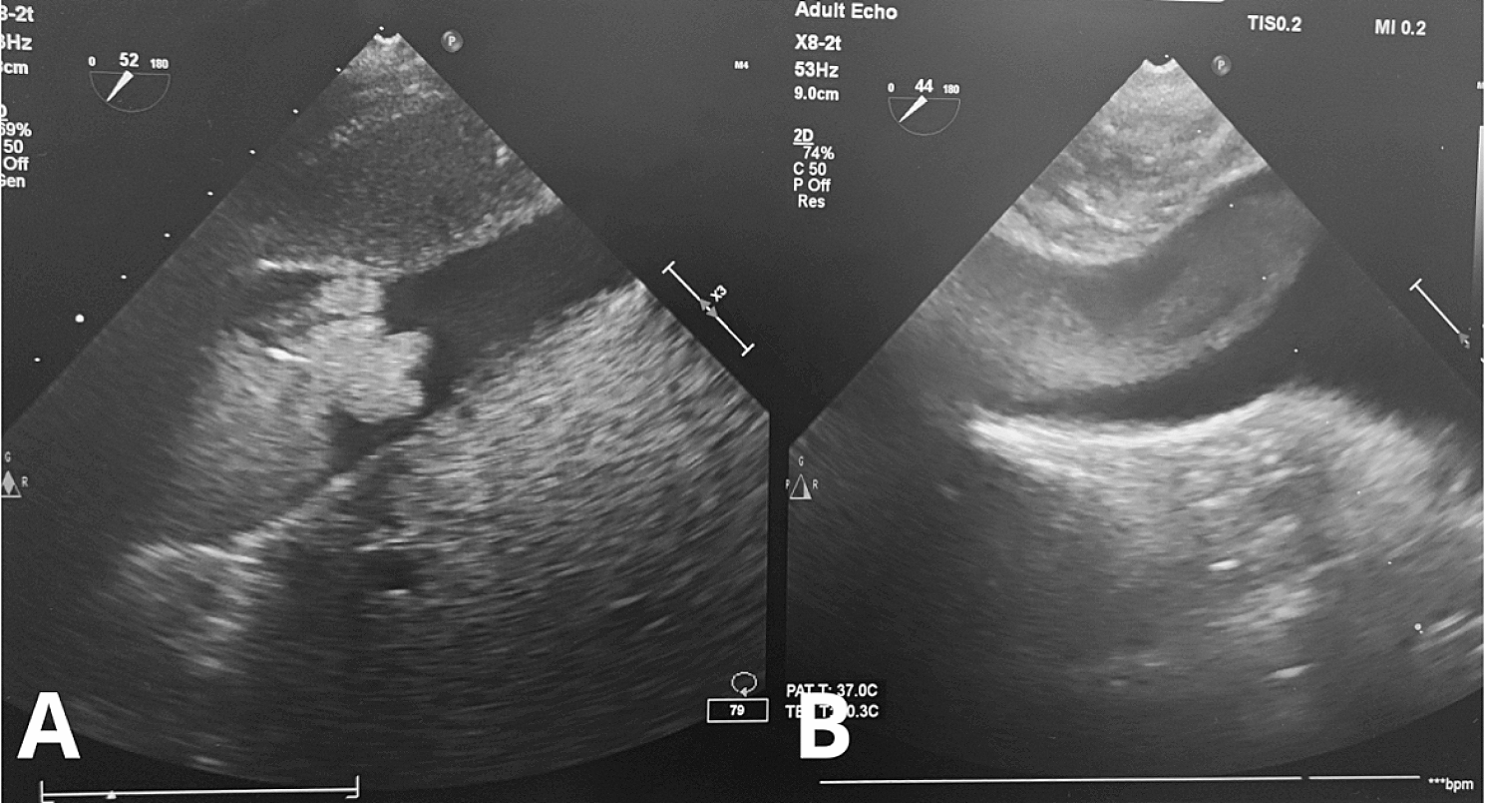
(**A**) TEE monitoring at the start of the surgery with TT level IIIc; (**B**) TT “uncoiled” into the right atrium after division of the IVC ligament

TEE showed that the TT was mobile inside the IVC and the right atrium (Fig. 2). Due to the newly detected extension of the TT into the right atrium, the central diaphragm tendon was dissected to the supra-diaphragmatic area, and the intra-pericardial IVC was identified. The right and left inferior phrenic vein were stapled during dissection. The posterior surface of the IVC was dissected and lumbar veins were stapled in order to facilitate circumferential control of the IVC. Hepatic hilum was isolated to allow a Pringle maneuver when needed and a replaced left hepatic artery was also identified. Dissection of the right kidney and right adrenal gland was completed and the kidney was left with a sole attachment to the IVC by the renal vein.

**Fig. 3:**
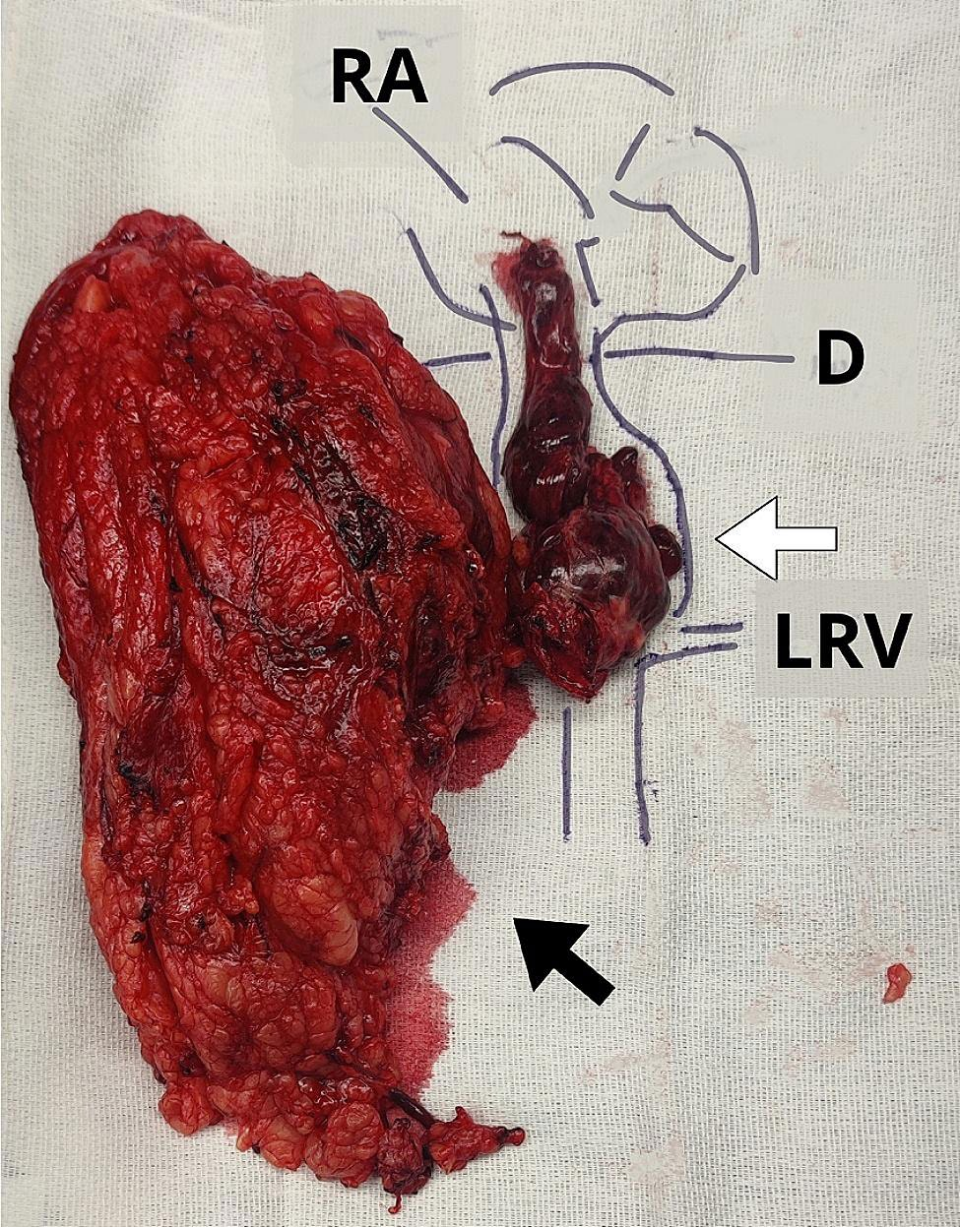
Pathology specimen of the right kidney (black arrow) showing the tumor thrombus all the way to the right atrium (white arrow). RA: right atrium; D: diaphragm; LRV: left renal vein

Under TEE monitoring vascular clamps were placed. First, a vascular clamp was placed in the infra-renal IVC. Then, the left renal vein was clamped and a Pringle maneuver was performed to temporarily occlude blood inflow into the liver. Finally, the replaced left hepatic artery was also clamped. We do not rutinely use heparin during clamping when the procedure is performed without cardiopulmonary bypass. After clamping, the IVC was opened from the right renal vein to below the major hepatic veins. The TT was pulled down to the infrahepatic IVC and taken out through the opening in the distal portion of the IVC, allowing for repositioning of the vascular clamp below the major hepatic veins. During this maneuver, blood outflow did not obscure the operative field due to complete isolation of the IVC and placement of the vascular clamp at the IVC below the major hepatic veins. At this time, the Pringle maneuver was discontinuedand blood flow to the liver was re-established. The Pringle maneuver lasted 4 min. The TT was removed en bloc along with the right kidney and a segment of the IVC (Fig. 3). Continuous TEE monitoring guaranteed that the TT had not been dislodged during its extraction. Afterwards, IVC incision was closed with a 4/0 polypropylene suture in a running fashion.

In this case, the TT was not adherent to the IVC wall and vascular resection was not required, thus avoiding artificial prosthesis (PFTE or teflon) placement. In case of complete chronic obstruction of the IVC with extensive collateral circulation, we would have considereded cavectomy without reconstruction as an option. This technique has been proven to be safe and to have no significant effect in postoperative renal function. The cavectomy is usually performed with a mechanic stapler and oversuture of the IVC proximally and distally [18–19].

IVC filter was placed below the left renal vein to prevent postoperative bland thrombus embolism.

At the end of the surgery, a final TEE was performed to ensure no pulmonary artery emboli or TT were present. Blood loss and packed red blood cell (PRBC) transfusions were 1200cc and 4U of PRBC, respectively. Pathology examination revealed 7.4 cm clear cell RCC Fuhrman grade 3 and lymph nodes were negative for carcinoma (pT3cN0Mn/a). The right adrenal gland was free of tumor. The patient was discharged home on post-operative day 4. The patient had an uneventful recovery with a creainine level of 1.4 mg/dL at 1-month follow-up. The patient is followed by both surgery and oncology teams.

## Discussion and conclusions

Currently, surgical excision remains the gold standard treatment option for patients with non-metastatic RCC with TT, providing a 5-year disease-free survival of approximately 60% [6, 20–21]. However, achieving complete resection of the TT, in some cases, remains a significant challenge.

A critical element in preoperative workup is to determine the level of the TT in order to tailor the surgical approach to each individual case. Many classification systems have been created, but the most commonly used is the one described by Neves and Zincke [15]. Subsequently, Ciancio et al. [14] established a subclassification for level III tumor thrombi, dividing it into four groups: IIIa (retrohepatic IVC below major hepatic veins), IIIb (retrohepatic IVC reaching the ostia of major hepatic veins), IIIc (retrohepatic IVC and extending above major hepatic veins, but below diaphragm), and IIId (suprahepatic and supradiaphragmatic IVC, reaching intrapericardial IVC, but infra-atrial). In our case, preoperative workup and TEE at the start of the surgery showed a mobile tumor thrombus level IIIc. Given the importance of a multidisciplinary approach in these difficult cases, preoperative planning was thoroughly discussed with the cardiothoracic team. Finally, it was decided to utilize an exclusively abdominal approach without CPB based on extension and size of TT in preoperative CT and MRI. TEE was performed after anesthesia induction, confirming a mobile TT that could potentially allow for an abdominal extraction. Nevertheless, CPB equipment and cardiovascular surgery team were put on standby and continuous TEE monitoring was requested. To our knowledge, this is the first reported case of a TT “uncoiling” during surgery from level IIIc to level IV without TT rupture or embolization.

Preoperative work-up in RCC with TT typically includes contrast enhanced CT and MRI. These techniques present limitations such as the risk of motion-related artifact from cardiac movement and spread of tumor during the interval between imaging and surgery [22]. For this reason, intraoperative TEE utility for TT stage evaluation has been reported widely since the 1990s [23]. The main advantages of TEE monitoring include enabling real-time visualization of the cephalad extension of the TT prior to incision and any changes it may suffer during surgery, evaluation of TT mobility, and confirming complete TT excision [20, 24–26]. Additionally, it provides information on cardiac function and early identification of pulmonary emboli [27].The incidence of intraoperative embolization is approximately 1.5% overall, with increasing risk among higher level thrombi, and is associated with a 75% risk of mortality, as reported by Shuchet al. [28]. A case of cranial displacement of the TT from level IIIc to level IIId due to compression during mobilization of the right kidney has been reported [29]. It manifested as periods of transient severe hypotension without proof of massive blood losss or pulmonary embolism. TEE images showed an obstruction of the IVC due to migration of the TT into the IVC right atrium junction. Surgical traction on the kidney relieved the IVC obstruction and surgery was completed without further incidents.

Another case that highlights the utility of TEE was reported by Erdemliet al. [30]. A 70-year old man with a RCC underwent transthoracic echocardiography two days prior to surgery, which showed a dense mass in the IVC that did not reach the right atrium. Therefore, an exclusive abdominal approach with infrahepatic clamping was planned. After anesthesia induction, intraoperative TEE unexpectedly revealed two tumor thrombi (one fixed and one free-floating) in the right atrium. These findings prompted a change in surgical approach and surgery was successfully completed under CPB.

In conclusion, preoperative workup and tumor staging is fundamental for surgical planning, although the surgeon must be prepared for unexpectedintraoperative events that may require a change in surgical approach. For this reason, TEE is critical for intraoperative continuous monitoring of TT level.

## Data Availability

The data presented in this article are available from the corresponding author on reasonable request.

## Abbreviations

CPB: cardiopulmonary bypass
IVC: inferior vena cava
RCC: renal cell carcinoma
TEE: transesophageal echocardiography
TT: tumor thrombus
